# Risk of Breast Cancer Revealed by Mammographic Screening in Czech Women Aged 40–45 Years, a Monocentric Cohort Study

**DOI:** 10.3390/diagnostics10090726

**Published:** 2020-09-21

**Authors:** Lívia Večeřová, Marek Petráš, Alexander M. Čelko, Jolana Rambousková

**Affiliations:** 1 Third Faculty of Medicine, Charles University, Ruská 87, 10000 Prague, Czech Republic; livia.vecerova@bulovka.cz (L.V.); martin.celko@lf3.cuni.cz (A.M.Č.); jolana.rambouskova@lf3.cuni.cz (J.R.); 2 Bulovka Hospital, Budínova 67/2, 18081 Prague 8, Czech Republic

**Keywords:** mammography, mass screening, diagnostic imaging, women’s groups, breast cancer risk

## Abstract

The aim of the present study was to evaluate breast cancer risk in women aged 40–45 years not included in the routine mammographic screening programme in the Czech Republic and to assess the suitability of the screening interval. Our cohort study was conducted using registry data of one mammography centre (Bulovka Hospital in Prague) between 1 January 2008 and 31 December 2017. The risk of breast cancer was evaluated using a positive predictive finding (PPF) corresponding to the Breast Imaging-Reporting and Data System (BI-RADS) scores of 4 and 5. The annual PPF incidence rate achieved 2.25 per 1000 women aged 40–45 years and was not significantly different from that (3.31) of women of 45–50 years of age as demonstrated by an adjusted hazard ratio of 0.75 (95% confidence interval: 0.42–1.33). It was found that a screening interval longer than 3 years increased the chance of PPF occurrence 1.7 times independently of the women’s age, signalling a risk of failure of early detection of breast cancer. The same PPF incidence rates both in women aged 40–45 years and in older ones indicates that even younger women should be eligible for enrolment in the routine mammographic screening programme in the Czech Republic.

## 1. Introduction

Mammographic screening as a routine preventive examination to reduce breast cancer mortality of women has been introduced in developed countries since the 1990s [1]. However, it was only in the first decade of the 21st century that the programme was actually implemented in most of European countries. In 2018, the screening helped to reveal 5.0% and 6.1% of the cumulative risk of malignant breast cancers in women worldwide and Central plus Eastern Europe combined, respectively [2].

In the Czech Republic, a nationwide programme of mammographic screening was launched in September 2002. The current network of 72 mammography centres ensures routine screening of all Czech women aged over 45 years irrespective of their place of residence. Under the project, the results of verified mammographic findings from all centres were compulsorily collected at the Institute of Biostatistics and Analyses of the Faculty of Medicine of Masaryk University, Brno, Czech Republic, twice a year. After rigorous validation, the data were entered into a central database [3].

Our study aimed to evaluate the incidence rate of suspicious abnormalities and findings highly suggestive of malignancies of the breast including cases of extremely low-risk malignancy as determined by mammographic screening in one centre (Bulovka Hospital, Prague, Czech Republic). We sought to determine whether these incidence rates differ between women aged 40–45 years with no routine screening and those older than 45 years undergoing a regular mammographic examination. We considered a positive finding more appropriate than confirmed breast cancer to recognize early risk of breast cancer in younger women. Furthermore, we tried to evaluate the current two-year screening interval against a longer one based on screening age.

## 2. Materials and Methods

In the Czech Republic, a nationwide mammography screening programme was launched in September 2002 with central data processing not started until 2004. The examination is offered every two years to all female residents aged 45+ with no upper age limit. Moreover, the prevention programmes run by local health insurance companies enable voluntary mammography examinations for women aged 40–45 years.

**Figure 1:**
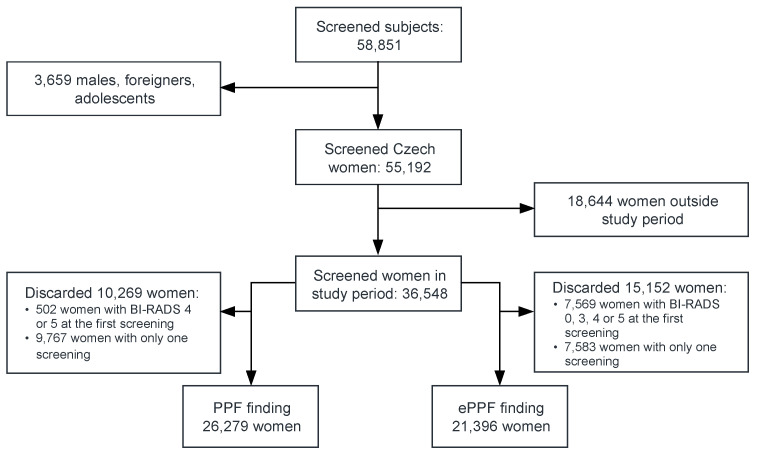
Study population flow.

We conducted a retrospective cohort study entitled MAMOCO (MaMmographic Cohort study) based on data obtained from the local registry of one screening centre (Bulovka Hospital in Prague). The study was approved by the Ethics Committee of the Bulovka Hospital (registration number: 29.11.2019/9368/EK-Z). Eligible for inclusion in the study were all adult women of Czech nationality attending at least one screening visit from 1 January 2008 to 31 December 2017 irrespective of any previous screening (Figure 1). All recruited women lived in the neighbourhood of the screening centre.

The outcome mammography as a basic screening method was assessed using the Breast Imaging-Reporting and Data System (BI-RADS) [4] determined independently by two trained radiologists or, possibly, by a third one if consensus had not been achieved. A finding predicting low-to-high probability of a malignancy was classified as BI-RADS 4 or 5 and was further referred to as a positive predictive finding (PPF) that subsequently needed tissue diagnosis. A finding classified as BI-RADS 3 should reflect a very or an extremely low risk of a malignancy requiring re-examination within a short follow-up period. A BI-RADS score of 0 identified an incomplete outcome with an unclear likelihood of cancer thus necessitating an additional imaging test. Women with BI-RADS scores of 1 and 2 were assessed as a negative and benign finding, respectively, and were subsequently referred for only routine screening. All outcomes requiring further examination, i.e., BI-RADS 0, 3, 4 and 5, were assessed as an extended positive predictive finding (ePPF) defined as a secondary endpoint of the analysis.

All women aged 40–70 years were stratified into 5-year age cohorts while those older than 70 years were assessed in one cohort. A control cohort included women aged 45–50 years. Women with a PPF or an ePPF at the first mammography screening as well as those with only one screening within the study period were excluded from the current analysis.

The incidence rates of a PPF (IR-PPF) and an ePPF (IR-ePPF) were expressed per 1000 person-year (py) and calculated for all age groups. Any change in these rates was investigated using an adjusted hazard ratio (aHR) with a p-value obtained from Cox regression at a level of significance set at 0.05. The power of result was verified with an exponential test comparing two independent IR-PPFs, i.e., in the tested cohort of women aged 40–45 years and the control one aged 45–50 years. The assumption was that the sample size of cohorts would be justified at an estimated power higher than 80%.

The screening intervals were stratified as follows: ≤24 months, >24–30 months, >30–36 months and >36 months. The attack rates of PPF and ePPF were determined for women stratified into 10-year groups within each interval group. A total of 100,768 records were used with the screening interval evaluated independently of the number of examinations each woman had had within the study period. To assess whether a prolonged screening interval for each age group could increase the PPF attack rate, an odds ratio (OR) calculated using logistic regression was used. A reference group of women aged 40-50 years, screened at a 24–30-month interval, was created. For the purpose of this analysis, the level of significance was set at 0.20 as it was required to increase the sensitivity of the method to rule out any potential underestimation of the attack rate.

Continuous data were summarized using standard descriptive statistics, i.e., mean including standard deviation. Categorical variables were presented as relative frequencies complemented with 95% confidence interval. Statistical analyses and regressions were performed using STATA version 15.1 software (StatCorp. Lakeway Drive, TX, USA).

When applying the Newcastle–Ottawa Quality Assessment Scale (NOS) to our cohort, the study was awarded a total of 7 stars (selection: 4, comparability: 1 and outcome: 2) [5].

The study cohort was representative of 58.4% women in the community with all age cohorts coming from the same community. In our study, eligible for enrolment were only women with a negative baseline mammographic finding. Comparability of cohorts by both sex (only women) and residence was similar. Assessment of the outcome was performed using a registry of records validated by the Institute of Biostatistics and Analyses. Follow-up duration was at least 2 years. The number of subjects lost to follow-up was not higher than 20%.

## 3. Results

During the study period, 36,548 women participated in mammographic screening programme, i.e., screening rate of 58.44%. The number of women attending their first-ever screening examination decreased from 8074 in 2008 to 1524 women in 2017 (Table 1). The median of age of women was 57.60 years at the first screening and they were followed up for a median of 4.31 years. The mean age of new attendees dropped significantly from 57.70 to 46.65 years between the first and last years of the study.

The lowest screening rate of 7.35% was achieved in women aged 40–45 years having only voluntary mammographic examination covered by their health insurance companies. The routine screening rates for each 5-year age cohort of women aged 45–70 years were 63.83%, 86.28%, 78.24%, 72.53% and 72.33%. A routine mammographic screening examination was attended by only 49.79% of the oldest women aged 70+.

The incidence rates of PPF and ePPF were assessed in sets of 26,279 and 21,396 women, respectively, who had had an initial negative finding and more than one screening (see Figure 1). Total PPF prevalence was low at the first study screening achieving 1.37% (95% CI: 1.26–1.50%). Conversely, BI–RADS scores of 0 and 3 were found in 4.88% (95% CI: 4.66–5.10%) and 14.5% (95% CI: 14.1–14.8%) of women, respectively, during the first screening.

The incidence rate of PPF in women aged 40–45 years achieved 2.25 per 1000 py, even if the annual rate in the control cohort was higher, i.e., 3.31 per 1000 py, an aHR of 0.75 (95% CI: 0.42–1.33), as well as the absence of statistical significance indicated no lower PPF occurrence in younger women (Table 2). The power test value of 81.3% indicated that the incidence rates in both age cohorts were similar, and the sample size was sufficient to demonstrate this result. The incidence rate of a PPF was stable among the age groups from 40 to 65 years, not exceeding 3.51 per 1000 py. Conversely, this rate achieved 4.59 per 1000 py in women over 65 years of age and was significantly higher than that in women of the control cohort as demonstrated by the aHR (1.34; 95% CI: 1.01–1.76).

The IR-ePPF was 5–18-fold higher than the IR-PPF depending on the age group. Despite this, a similar outcome was found for IR-ePPF in the tested age group because the aHR (1.15; 95% CI: 0.97–1.37) did not show any different incidence rates between the tested and control cohorts. Moreover, ePPF occurrence was more frequent in younger women aged 40–45 years (IR-ePPF = 42.44 per 1000 py) compared to the compared to the other cohorts. The ePPF outcome was more often reported in women aged 40–50 years compared to the older ones.

A total of 78.22% (95% CI: 77.90–78.54%) of mammographic re-examinations was done within 6 months of the 2-year interval. A further 10.36% (95% CI: 10.12–10.60%) was performed within 30–36 months of the preceding screening and 9.75% (95% CI: 9.52–9.98%) later than 3 years. Only 1.68% (95% CI: 1.58–1.78%) of the screening examinations was performed earlier than 2 years.

The PPF attack rate was 0.75% (95% CI: 0.68–0.83%) and, at 2 years after their preceding examination, it remained similar in women less than 70 years of age. This rate was stable for 12 months because it did not differ between both screening intervals of 24–30 and 30–36 months. No significant difference in the PPF attack rate for intervals shorter than 36 months was shown among these age-stratified women as demonstrated by p–values >0.20 and 80% confidence intervals of the OR (Table 3). Conversely, the PPF attack rate increased to 1.38% (95% CI: 1.10–1.72) in these women if their screening interval exceeded 3 years. An OR of 1.73 (95% CI: 1.36–2.18) indicated a 73% increase in the PPF attack rate in women aged >40 years screened at intervals longer than 3 years, irrespective of their age.

Women older than 70 years had a PPF attack rate of 1.24% (95% CI: 0.88–1.69%) at 24–30 months after their preceding examination. The rate was significantly higher at both levels of significance of 0.20 and 0.05, compared to reference women, i.e., OR = 1.72 (95% CI: 1.34-2.21). However, at intervals longer than 30 months, their PPF occurrence did not differ from that of the reference group.

The ePPF attack rate, especially with a BI-RADS of 3, did not vary by the screening interval among women aged 40–50 years. In women older than 50 years, ePPF occurrence was found to be significantly lower compared to that of the reference group at any screening interval. Moreover, the ePPF attack rate declined with increasing age.

## 4. Discussion

We focused only on the outcomes of routinely performed basic screening with no optional examinations that increase recall and cost rates. Our cohort study results clearly demonstrated that routine mammographic screening extended to women aged 40–45 years could markedly contribute to early detection of breast cancer. While the incidence rate of suspicious abnormalities and findings highly suggestive of a malignancy was lower in these women compared to the older ones, the risk of developing breast cancer was not different as confirmed by the hazard ratio. This outcome was in line with previously published findings [6,7,8,9]. Therefore, some countries decided to include women aged 40–45 years in their mammographic screening programmes [6,10].

A higher interception of BI-RADS 4 or 5 was found in women older than 65 years, especially in those over 70 years of age. This can be explained by the consistently high density of the breast gland in women receiving hormone replacement therapy [11,12] and age-specific risk factors such as overweight or obesity and low physical activity.

It was shown that women aged 40–50 years were more often classified as BI-RADS 0 and 3. An additional imaging test and/or records of a prior examination, including a re-examination within a shorter interval of 6 months to complete the final assessment, was required more often in younger than older women. This may have been due to later reduction of the gland in younger women leading to high mammographic density reducing the sensitivity of imaging in which case the finding had to be verified by a further examination. As the number and type of examination techniques may vary among countries, this could lead to either a false positive or a false negative outcome [13,14,15,16]. The ambiguity of any finding could also be influenced by other predictors such as full-time work, oral contraceptive use and other behaviour patterns contributing to higher mammographic density [17].

A screening examination performed every 2 years was suitable for women below 70 years of age because the PPF occurrence among women stratified into 10-year cohorts was consistent without any significant increase. Surprisingly, suspicious or highly suggestive cases were more often observed within the 24–30-month interval for women older than 70 years likely linked with age, which is considered a major risk factor for late-age breast cancer [18]. To date, no consensus has been reached regarding the upper limit for mammography screening. The incidence of breast cancer increases gradually with increasing age and, without screening, breast cancer in older women goes more often undiagnosed until an advanced stage [19,20].

Irrespective of age, the present study confirmed that a screening interval longer than 3 years was associated with an increased number of suspicious abnormalities and findings highly suggestive of breast cancer. The implication is that a delayed examination could lead to failure to detect breast cancer at an early stage; hence, every effort should be made not to exceed the 3-year screening interval.

The occurrence of a probably benign finding or a finding requiring an additional imaging test declined with increasing age of women, irrespective of the screening interval investigated. It seems that, after menopause, a large part of the glandular tissue of the breast is replaced by fat thus facilitating mammography-based detection of breast cancer [21].

A potential limitation of the present cohort study could be its monocentric design that did not enable us to compare our outcomes with those obtained in another centre. Nevertheless, it could be assumed that the study results should be generally applicable to the Czech target population as demonstrated by the higher NOS score of a high-quality study. Another limitation could be the low screening rate of women aged 40–45 years that contributes to potential bias. However, the outcomes should not be influenced by this fact because the power analysis demonstrated a sufficient sample size of the youngest cohort. Finally, the routine screening rate, ranged between 50% and 86% of women depending on the age cohort, was possibly reflected in the higher incidence rates of a PPF in the oldest women because only 50% of them participated in the routine mammographic screening programme.

## 5. Conclusions

In conclusion, not only the similar incidence rate of suspicious abnormalities and findings highly suggestive of breast cancer but also the more frequent interception of probably benign forms indicates why women aged 40–45 years should be considered for enrolment in the routine mammography screening programme in the Czech Republic. An additional argument is the fact that younger women have more often poorly differentiated lcarcinoma of the breast, or high-grade ductal carcinoma in situ as well as triple negative breast carcinoma. Nevertheless, the success of a screening programme is dependent on early detection of any positive predictive finding not only in younger women but also on strict adherence to a <3-year screening interval in all women.

## Figures and Tables

**Table 1:** Number of women enrolled in the study, including mean age of the first study screening and mean follow-up.

Year of 1st Study Screening	N ^1^	Median of Age (IRQ) ^2^ (Years)	Median of Follow-up (IRQ) (Years)	Baseline Finding (1st Screening)					
				BI-RADS 4 or 5		BI-RADS 3		BI-RADS 0	
				*n* ^3^	Prevalence (95% CI ^4^)	*n*	Prevalence (95% CI)	*n*	Prevalence (95% CI)
**2008**	8074	57.70 (11.30)	8.22 (3.04)	65	0.81 (0.62–1.02)	1052	13.03 (12.30–13.78)	376	4.66 (4.21–5.14)
**2009**	7422	58.60 (11.70)	6.74 (2.38)	71	0.96 (0.75–1.21)	885	11.92 (11.20–12.68)	209	2.82 (2.45–3.22)
**2010**	3783	59.60 (17.90)	5.92 (4.04)	46	1.22 (0.89–1.62)	635	16.79 (15.61–18.02)	141	3.73 (3.15–4.38)
**2011**	2846	59.40 (21.20)	4.36 (3.84)	58	2.04 (1.55–2.63)	467	16.41 (15.07–17.82)	94	3.30 (2.68–4.03)
**2012**	3436	59.00 (19.80)	4.12 (2.41)	65	1.89 (1.46–2.40)	528	15.33 (14.18–16.62)	183	5.33 (4.60–6.13)
**2013**	3400	55.90 (20.80)	2.43 (4.12)	43	1.26 (0.92–1.70)	633	18.62 (17.32–19.97)	190	5.59 (4.84–6.41)
**2014**	2480	55.80 (21.20)	2.11 (2.43)	46	1.85 (1.36–2.47)	450	18.15 (16.65–19.72)	160	6.45 (5.52–7.49)
**2015**	2042	51.60 (21.90)	0.00 (2.13)	46	2.25 (1.65–2.99)	313	15.33 (13.79–16.96)	201	9.84 (8.59–11.22)
**2016**	1541	47.80 (20.50)	0.00 (0.00)	24	1.56 (1.00–2.31)	198	12.85 (11.22–14.62)	164	10.64 (9.15–12.29)
**2017**	1524	46.65 (17.30)	0.00 (0.00)	38	2.49 (1.77–3.41)	124	8.14 (6.81–9.62)	64	4.20 (3.25–5.33)
**Total**	36,548	57.60 (17.00)	4.31 (6.97)	502	1.37 (1.26–1.50)	5285	14.46 (14.10–14.83)	1782	4.88 (4.66–5.10)

^1^ N, number of women; ^2^ IRQ, interquartile range; ^3^ n, number of initial baseline findings; ^4^ CI, confidence interval.

**Table 2:** Incidence rates of PPFs and ePPFs and adjusted hazard ratio including the 95% confidence interval.

Age Group (Years)	PPF ^1^					ePPF ^2^				
	N ^3^	py ^4^	IR-PPF ^5^ (per 1000 py)	aHR ^6^ (95% CI ^7^)	*p*	N	py	IR-ePPF ^8^ (per 1000 py)	aHR (95% CI)	^10^ *p*
>40–45	13	5776	2.25 (1.31–3.88)	0.75 (0.42–1.33)	0.316	161	3794	42.44 (36.36–49.83)	1.15 (0.97–1.37)	0.096
>45–50	105	31,696	3.31 (2.74–4.01)	1 ^9^	x	806	20,996	38.39 (35.83–41.13)	1 ^9^	x
>50–55	83	23,475	3.54 (2.85–4.38)	0.94 (0.70–1.25)	0.657	529	17,671	29.94 (27.49–32.60)	0.74 (0.67–0.83)	<0.001
>55–60	98	28,663	3.42 (2.80–4.17)	0.92 (0.70–1.22)	0.577	560	22,921	24.43 (22.49–26.54)	0.61 (0.55–0.68)	<0.001
>60–65	94	32,215	2.92 (2.38–3.57)	0.80 (0.60–1.06)	0.114	617	26,617	23.18 (21.42–25.08)	0.58 (0.52–0.64)	<0.001
>65–70	93	20,281	4.59 (3.74–5.62)	1.34 (1.01–1.76)	0.043	364	17,149	21.23 (19.15–23.52)	0.54 (0.48–0.61)	<0.001
>70	45	9598	4.69 (3.50–6.28)	1.73 (1.21-2.45)	0.002	157	8284	18.95 (16.21–22.16)	0.52 (0.43–0.61)	<0.001

^1^ PPF, positive predictive finding; ^2^ ePPF, extended positive predictive finding; ^3^ N, number of positive mammographic outcome; ^4^ py, person-year; ^5^ IR-PPF, incidence rate of a PPF; ^6^ aHR, adjusted hazard ratio from COX regression; ^7^ CI, confidence interval; ^8^ IR-ePPF, incidence rate of an ePPF; ^9^ control cohort; ^10^ *p*, *p*-value from COX regression.

**Table 3:** Attack rates of PPFs and ePPFs and odds ratio to the reference group for each age and interval groups.

Age Group (Years)	Screening Interval (Months)	PPF ^1^				ePPF ^2^			
		N ^3^	Attack Rate (95% CI ^4^)	OR ^5^ (80% Cl)	*p* ^6^	N	Attack Rate (95% CI)	OR (80% Cl)	*p*
>40–50	≤24	392	0.77 (0.16–2.22)	1.06 (0.50–2.25)	0.923	230	10.43 (6.80–15.13)	1.03 (0.78–1.36)	0.905
	>24–30	11,749	0.72 (0.58–0.89)	1		7004	10.17 (9.47–10.90)	1	
	>30–36	1889	0.58 (0.29–1.04)	0.80 (0.53–1.21)	0.497	1141	10.69 (8.96–12.63)	1.06 (0.93–1.21)	0.624
	>36	1658	1.15 (0.69–1.78)	1.59 (1.15–2.21)	0.069	1003	10.87 (9.01–12.96)	1.08 (0.94–1.24)	0.537
>50–60	≤24	503	1.19 (0.44–2.58)	1.66 (0.96–2.85)	0.235	335	10.45 (7.39–14.23)	1.03 (0.82–1.30)	0.88
	>24–30	17,251	0.77 (0.64–0.91)	1.06 (0.88–1.27)	0.686	12,568	6.82 (6.38–7.27)	0.65 (0.60–0.69)	<0.001
	>30–36	2116	0.80 (0.47–1.28)	1.11 (0.79–1.56)	0.692	1535	6.32 (5.15–7.65)	0.60 (0.52–0.69)	<0.001
	>36	2110	1.23 (0.81–1.80)	1.71 (1.28–2.28)	0.017	1547	6.46 (5.29–7.81)	0.61 (0.53–0.70)	<0.001
>60–70	≤24	155	0.00 (0.00–2.35)	–	-	119	1.68 (0.20–5.94)	0.15 (0.06–0.38)	0.012
	>24–30	17,523	0.79 (0.67–0.94)	1.10 (0.92–1.31)	0.539	13,898	5.62 (5.24–5.94)	0.53 (0.49–0.56)	<0.001
	>30–36	2116	0.61 (0.33–1.05)	0.85 (0.58–1.24)	0.568	1689	5.15 (4.15–6.32)	0.48 (0.41–0.56)	<0.001
	>36	2020	1.73 (1.21–2.40)	2.42 (1.87–3.13)	<0.001	1623	6.84 (5.66–8.18)	0.65 (0.57–0.74)	<0.001
**>70**	≤24	13	0.00 (0.00–24.71)	–	-	12	0.00 (0.00–26.46)	–	-
	>24–30	3073	1.24 (0.88–1.69)	1.72 (1.34–2.21)	0.007	2542	4.92 (4.11–5.83)	0.46 (0.40–0.52)	<0.001
	>30–36	447	0.89 (0.24–2.28)	1.24 (0.64–2.39)	0.689	381	4.46 (2.62–7.05)	0.41 (0.30–0.57)	0.001
	>36	392	0.77 (0.16–2.22)	1.06 (0.50–2.25)	0.934	327	4.59 (2.59–7.45)	0.42 (0.30–0.60)	0.003

^1^ PPF, positive predictive finding; ^2^ ePPF, extended positive predictive finding; ^3^ *N*, number of women; ^4^ CI, confidence interval; ^5^ OR, odds ratio, ^6^ *p*, *p*-value from logistic regression.

## Abbreviations

aHR	adjusted hazard ratio
BI-RADS	Breast Imaging-Reporting and Data System
CI	confidence interval
ePPF	extended positive predictive finding
IR-ePPF	incidence rate of an ePPF
IR-PPF	incidence rate of a PPF
NOS	Newcastle–Ottawa Quality Assessment Scale
OR	odds ratio
PPF	positive predictive finding
py	person–year
